# 1.25 Å resolution structure of an RNA 20-mer that binds to the TREX2 complex

**DOI:** 10.1107/S2053230X1501643X

**Published:** 2015-09-23

**Authors:** Eugene Valkov, Murray Stewart

**Affiliations:** a MRC Laboratory of Molecular Biology, Francis Crick Avenue, Cambridge Biomedical Campus, Cambridge CB2 0QH, England

**Keywords:** RNA, noncanonical base pair

## Abstract

The 1.25 Å resolution *H*32:*R* crystal structure of a 20 nt ribonucleotide that binds to the TREX-2 complex with high affinity shows a double-stranded RNA duplex arranged along a crystallographic 3_1_ axis in which the antiparallel chains overlap by 18 nucleotides and are related by a crystallographic twofold axis. The duplex shows C–A, U–U and C–C noncanonical base pairings together with canonical Watson–Crick A–U and G–C pairs and a G–U wobble.

## Abstract 2

The 1.25 Å resolution crystal structure of a 20 nt ribonucleotide that binds to the TREX2 complex with high affinity shows a double-stranded RNA duplex that contains C–A, U–U and C–C noncanonical base pairings together with canonical Watson–Crick A–U and G–C pairs and a G–U wobble.

## 1. Introduction   

The yeast TREX2 complex functions to integrate the nuclear components of the gene-expression pathway with mRNA nuclear export as well as localizing actively expressing genes such as *GAL1* to nuclear pores (NPCs). TREX2 is based on a Sac3 scaffold, to which Thp1, Sem1, Cdc31 and two chains of Sus1 bind. The complex can be divided into three regions, each of which has been characterized structurally and functionally: (i) the CID region, containing Cdc31, both Sus1 chains and Sac3 residues 727–805 (Jani *et al.*, 2009 ▸), that interacts with nuclear basket components such as Nup1 and, in addition to facilitating mRNA export, functions to localize actively transcribing genes to NPCs (Jani *et al.*, 2014 ▸), (ii) the N-terminal region of Sac3 (residues 1–140) that binds to the principal yeast mRNA nuclear export factor, Mex67–Mtr2 (Dimitrova *et al.*, 2015 ▸), and (iii) the M-region, in which Thp1 and Sem1 bind to Sac3 residues 250–563 and which contains two juxtaposed winged-helix domains that bind RNA (Ellisdon *et al.*, 2012 ▸). Previous work indicated a level of specificity in RNA binding by TREX2 in that polyuridine binds more strongly than other polyribonucleotides (Ellisdon *et al.*, 2012 ▸), and studies on the human analogue of TREX2 that is based on GANP (the Sac3 homologue) also showed some specificity in binding to a subset of transcripts (Wickramasinghe *et al.*, 2014 ▸). Here, we report the 1.25 Å resolution crystal structure of a 20 nt RNA fragment identified as having a higher affinity for TREX2 than polyuridine.

## 2. Methods and materials   

An RNA oligonucleotide with the sequence ACCUGAGU­UCAAUUCUAGCG was synthesized and HPLC-purified by Integrated DNA Technologies (Interleuvenlaan, Belgium) and dissolved at a concentration of 1.0 m*M* in 10 m*M* Tris–HCl pH 7.4. No further steps involving thermal denaturation and cooling were taken to promote duplex formation and additional magnesium chloride was not added to the sample. Crystallization conditions were screened using sparse-matrix formulations at 18°C by sitting-drop vapour diffusion using ∼200 nl drops. Crystals were observed in many conditions after several days. A variety of morphologies including needles, clusters and large individual crystals were observed in PEG-based (PEG 2K MME and PEG ranging from 600 to 8K) conditions and conditions where inorganic salts (ammonium sulfate, lithium sulfate, sodium chloride) were the main precipitant and over a range of different pH values (ranging from 5.5 to 10.5). No further optimization from initial screening was carried out. For X-ray data collection, crystals were supplemented with 15–30%(*v*/*v*) glycerol prior to vitrification in liquid nitrogen.

**Figure 1:**
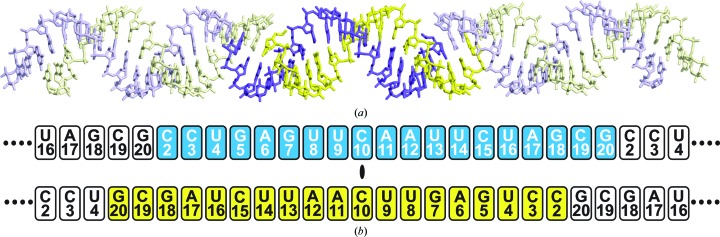
Generation of an RNA duplex by crystallographic symmetry. (*a*) In the *H*32:*R* crystal, the single chain in the asymmetric unit (blue) and its symmetry mate (yellow) related by a crystallographic twofold axis generate a double-stranded antiparallel RNA duplex (highlighted in more saturated colours) in which the two chains in the duplex overlap by 18 ribonucleotides, as illustrated schematically in (*b*). C19 and G20 overlap with the next duplex to generate a continuous A-type RNA double helix along a crystallographic 3_1_ axis. In addition to the canonical base pairings C2–G18, A6–U14, U8–A12 and U9–A11, there is a classic wobble G7–U13 pair together with C3–A17, U4–U16 and C10–C10 noncanonical pairs.

**Figure 2:**
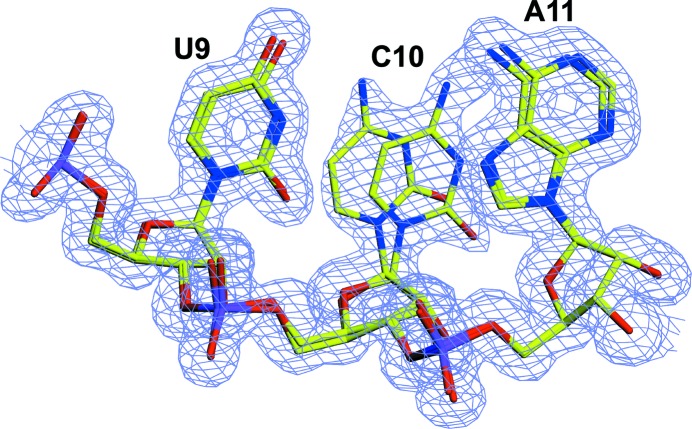
Alternate conformations adopted by ribonucleotides 9–11. The occupancies of these alternative conformations refined as 0.52 and 0.48, consistent with their being present in approximately equal proportions. The 2*m*|*F*_o_| − *D*|*F*_c_| composite OMIT map is contoured at 1σ.

Diffraction data were collected on beamline I04-1 at the Diamond Light Source, Didcot, England. Surprisingly, there was no clear correlation between crystal appearance and diffraction quality. Although almost all of the crystals tested produced powder diffraction patterns, one crystal grown in 2.0 *M* ammonium sulfate, 0.2 *M* sodium chloride, 0.1 *M* sodium cacodylate pH 6.5 (Table 1 ▸) diffracted to high resolution and data were collected using a φ increment of 0.5°. These crystals diffracted to 1.25 Å resolution, with unit-cell parameters *a* = 39.9, *b* = 39.3, *c* = 156.9 Å, α = β = 90, γ = 120° (Table 2 ▸). Owing to the possibility of twinning and the presence of noncystallographic symmetry, it was not possible to distinguish between *H*3:*R* and *H*32:*R* symmetry at this stage. Data were processed and reduced using *XDS* (Kabsch, 2010 ▸) and *AIMLESS* (Evans, 2011 ▸). Twinning tests for *H*3:*R* symmetry were equivocal using *phenix.xtriage* (Adams *et al.*, 2010 ▸) and thus it was thought prudent to initially attempt molecular replacement using *H*3:*R* symmetry. Conventional molecular replacement using models comprising helices and duplexes was unsuccessful, and thus a set of shell scripts was developed that used fragments of high-quality RNA models from the PDB together with several *ab initio* models to derive a series of low-scoring solutions in *Phaser* (McCoy *et al.*, 2007 ▸). All of the solutions obtained, irrespective of their scores, were subjected to 20 cycles of simulated annealing in Cartesian space as implemented in *phenix.refine* (Afonine *et al.*, 2012 ▸) and were then used as starting models for *phenix.autobuild* (Adams *et al.*, 2010 ▸) in a fully automated protocol. Model improvement was monitored using both *R*_free_ and correlation coefficients during successive rounds of density modification and reciprocal-space refinement. Several different models of highly truncated duplexes, comprising two and three Watson–Crick base pairs, converged to generate almost identical models with *R*_free_ < 40% and with clear features corresponding to bases and phosphate backbone in 2*m*|*F*_o_| − *D*|*F*_c_| electron-density maps together with features in the *m*|*F*_o_| − *D*|*F*_c_| difference density maps that were not yet accounted for by the model. The density obtained was sufficiently good to enable purines and pyrimidines to be assigned and the ribonucleotide sequence to be fitted unequivocally. In the crystals, nucleotides 2–20 of the RNA formed an antiparallel helical duplex (Fig. 1 ▸) with its axis coincident with a crystallographic 3_1_ screw axis and one of the twofold axes relating the two chains coincident with the position of a crystallographic twofold axis of the *H*32:*R* unit cell. Consequently, this symmetry was adopted for further processing. The density corresponding to cytosine 10 that was related by this twofold was unusual and appeared to be the result of alternative conformations adopted by this nucleotide that also slightly influenced the adjacent nucleotides (Fig. 2 ▸). The influence of the twofold axis perpendicular to the *c* axis on the distribution of diffraction intensities was very similar to that expected from (*h*, *k*, −*l*) twinning. Iterative cycles of rebuilding using *Coot* (Emsley *et al.*, 2010 ▸) and refinement with *phenix.refine* (Afonine *et al.*, 2012 ▸), introduction of anisotropic temperature factors and insertion of waters produced a model with an *R* factor of 17.9% and an *R*_free_ of 18.8% (Table 3 ▸). Some densities assigned to water probably represented ammonium ions that balanced the charge on the RNA phosphates but could not be identified unequivocally. The stereochemistry of the structure was assessed and validated with *MolProbity* (Chen *et al.*, 2010 ▸), which indicated that there were no bond-length/angle outliers, that all ribose puckers were canonical and that the clashscore of 1.43 was in the top 98% of structures determined at this resolution.

## 3. Results   

**Figure 3:**
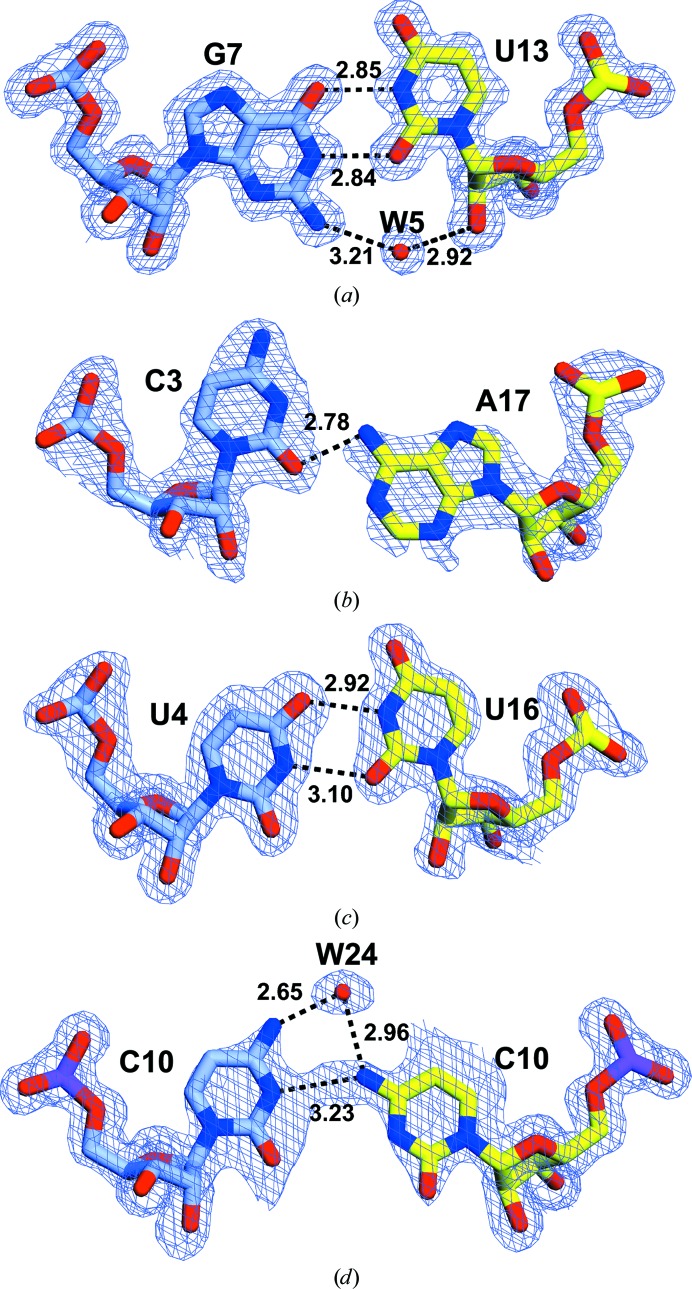
Noncanonical base pairings within the RNA duplex. (*a*) The G7–U13 wobble had the typical form supplemented by a water-bridged hydrogen bond involving guanine N2, water W5 and uridine ribose O2′. (*b*) The C3–A17 base pair was stabilized by a hydrogen bond between adenine N6 and cytosine O4, whereas the U4–U16 pairing (*c*) was based on hydrogen bonds between N3 and O4 of each base. (*d*) Cytosine 10 adopted alternate conformations; one of each is shown. The alternate conformations formed 3.2 Å amino–imino hydrogen bonds between N4 of one and N3 of the other, supplemented by putative hydrogen bonds between water W24 and N4 of both bases. The 2*m*|*F*_o_| − *D*|*F*_c_| composite OMIT map is contoured at 1σ and hydrogen-bond lengths are in Å.

The C2–G18, G5–C15, A6–U14, U8–A12 and U9–A11 base pairings in the duplex were all of the canonical Watson–Crick form and that of G7–U13 (Fig. 3 ▸ *a*) was of the canonical wobble form supplemented by a water-bridged hydrogen bond involving guanine N2, water 5 and uridine ribose O2′. In the similar C3–A17 wobble pairing the adenine N6 formed a single hydrogen bond to O4 of C3 (Fig. 3 ▸ *b*), which differs from the arrangement observed in the 2.5 Å resolution structure of a 16-mer duplex (Pan *et al.*, 1998 ▸), whereas the U4–U16 pairing (Fig. 3 ▸ *c*) was based on hydrogen bonds between N3 and O4 of each base. Cytosine 10 adopted alternate conformations, but both formed 3.2 Å amino–imino hydrogen bonds between N4 and N3 (Fig. 3 ▸ *d*) supplemented by hydrogen bonds between water 24 and N4 of both bases. A similar amino–imino hydrogen bonding was observed in the *Hepatitis C virus* internal ribosome entry site eIF3-binding site and thymidylate synthase mRNA, where multiple conformations of this base were also observed (Collier *et al.*, 2002 ▸; Tavares *et al.*, 2009 ▸). There was no clear electron density for adenine 1, which appeared to be disordered owing to its being displaced to facilitate the canonical C–G pairings between bases 19 and 20 in consecutive duplexes along the crystallographic 3_1_ axis. Consistent with this interpretation, the electron density for the ribose of cytosine 2 was also weak.

Although the RNA formed a duplex in the crystals, it appeared to be monomeric in solution and probably formed a hairpin using base pairing analogous to that observed in the duplex. The formation of duplexes from hairpins is favoured at the high ionic strength and RNA concentrations employed for crystallization (Nakano *et al.*, 2007 ▸).

## Supplementary Material

PDB reference: RNA 20-mer, 5c5w

## Figures and Tables

**Table 1:** Crystallization conditions

Method	Vapour diffusion
Plate type	Sitting-drop MRC 2-drop
Temperature (K)	291
RNA concentration	1.0m*M*
RNA solution	10m*M* TrisHCl pH 7.4
Reservoir solution	2.0*M* (NH_4_)_2_SO_4_, 0.2*M* NaCl, 0.1*M* sodium cacodylate pH 6.5
Volume and ratio of drop	200nl, 1:1
Volume of reservoir (l)	80

**Table 2:** Crystallographic data-collection statistics Values in parentheses are for the outer shell.

Diffraction source	Diamond Light Source, undulator beamline I04-1
Wavelength ()	0.9200
Detector	Pilatus 2M
Rotation range per image ()	0.5
Total rotation range	500
Exposure time per image (s)	0.15
Crystal-to-detector distance (mm)	175
Data-collection temperature (K)	100
Space group	*H*32:*R*
Unit-cell parameters (, )	*a* = *b* = 39.9, *c* = 156.87, = = 90, = 120
Resolution range ()	52.291.25 (1.281.25)
Unique reflections	13567
Total observations	319227
*I*/(*I*)	21.5 (1.6)
*R*_p.i.m._	0.021 (0.449)
*R*_merge_	0.100 (1.383)
Mean half-set correlation	1.000 (0.620)
Completeness (%)	98.6 (89.8)
Multiplicity	23.5
Wilson *B* factor (^2^)	10.5

**Table 3:** Refinement statistics

Non-H atoms	462
No. of water molecules	97
Bond-length deviation from ideal values ()	0.0076
Bond-angle deviation from ideal values ()	1.38
All-atom clashscore	1.43
Reflections used for the working set in refinement	12896
Random reflections assigned for cross-validation	667
*R*_work_/*R*_free_ (%)	17.9/18.8
